# The Third Institute for Cancer Vaccines and Immunotherapy (ICVI) symposium on immunotherapy: May 12–13, 2017, Royal Society, London, UK

**DOI:** 10.1007/s00262-018-2137-9

**Published:** 2018-03-02

**Authors:** Angus G Dalgleish

**Affiliations:** Institute for Infection and Immunity, St George’s University of London, Cranmer Terrace, London, SW17 0RE UK

**Keywords:** Immunotherapy, Symposium, Chemotherapy, Vaccines, Cyclophosphamide

## Introduction

This meeting was the third organised by the Cancer Vaccine Institute (CVI), now rebranded as the Institute for Cancer Vaccines and Immunotherapy (ICVI) to reflect the major input of non-vaccine-based immunotherapies in cancer treatment. (The previous meetings were held in 2013 and 2015, all at the Royal Society premises, Carlton Terrace, London.)

A major driver of these meetings has been to access the overall impact of immunotherapy in conjunction with other modalities, such as radiotherapy and other potentially ablative therapies, e.g. radiofrequency ablation (RFA), in addition to the potential for chemotherapy to synergise with immunotherapy, as opposed to the previous perception that they were mutually incompatible.

The hallmarks of cancer were set in stone in a definitive review by Hanahan and Weinberg, published in Cell in 2000. It did not include any reference to any immune influence or perhaps even more surprisingly to the clear effect of chronic inflammation on the development of many common cancers. This was eventually corrected in 2011 when the effects of tumour-promoting inflammation and its effects over genome instability and mutation, as well as the effects of downregulating cellular energetics and the extent tumours adapt to avoid immune destruction, were recognised. It was probably driven by the recognition of the first checkpoint inhibitor, ipilimumab (Yervoy) for melanoma, licensed by the FDA in the same year. Subsequently, the PD-1 checkpoint inhibitors, pembrolizumab (Keytruda), nivolumab (Opdivo), have now been licensed for not only melanoma but also lung cancer. In addition, activity has been documented for these agents in 23 different cancer types, including recent approvals for head and neck cancer and bladder cancer. However, in spite of what can only be described as a revolution in cancer treatment, response rates are still relatively low and relapses common. This has inspired a major focus of research based on biomarkers and a search for predictors of response. Much of this has focused on the expression of PD-1 and PD-L1 and the likelihood of a response to PD-1 inhibitors. Although a significant association was initially described, it is clear that in reality it is not that simple, with significant responses to PD-1 inhibitors being reported in PD-L1-negative individuals.

With this in mind, the symposium focused on how different immunotherapies could be combined with at least additive clinical benefit, although many of these combinations involve enhanced toxicities, such as when ipilimumab is combined with Nivolumab. A major focus was the potential for a real synergy with chemotherapy, particularly when given at lower doses, as well as other agents which exert subtle immune modulatory effects.

The symposium involved several references to chemotherapy agents, which when used at lower doses had a beneficial effect on the immune response, especially when cancer vaccines were used. A number of agents were mentioned, including gemcitabine, but the most frequently referred to agent was cyclophosphamide, although there was a considerable variation in the suggested doses and schedules used.

## Cyclophosphamide (CPA) enhanced immune protocols

**David Waxman** (Boston University, Boston, USA), focused on CPA mechanisms of action, particularly in its role in metronomic schedules. CPA depletes immune-suppressive T-reg cells and boosts cytokines that enhance the maturation of DCs as well as expand T cells with a memory phenotype. Using a murine glioma model, he showed that CPA given with a 6-day drug break activated innate immune activity which was more effective than more frequent delivery of CPA. After several cycles, long-term CD8+ T cells were induced, as well as activated NK cells. Non-responsive tumours were shown to lack IFN-αβ induction, suggesting that poly I:C treatment may help in correcting this deficiency.

It is important to note that the dose used for the 6-day cycle was given at 140 mg/kg, which, based on body surface area, corresponds to a human equivalent dose of 11.4 mg/kg. While this dose may seem high for humans, it is not high when considering the much more rapid elimination of cyclophosphamide in mice (*t*_1/2_ = 24 min in mice vs. *t*_1/2_ = 5 h in humans). Consistent with this difference in pharmacokinetics, there is a clear difference in optimal doses for cyclophosphamide immune effects in humans, where doses of 50–100 mg a day for 1 week on, followed by 1 week off, are able to suppress the T-reg response in patients. **David Waxman** also showed that cultivating autologous tumour cells with activated CPA, or with doxorubicin, can greatly enhance the immunogenicity of these cells when used as a vaccine. Several other speakers mentioned the beneficial use of CPA on T-reg function including **Emily Webb** (University of Southampton, Southampton, UK), **Sandra Tuyaerts** (Katholieke Universiteit (KU) Leuven, Leuven, Belgium), **Thomas Nesselhut** (Institute of Cell Therapy, Duderstadt, Germany) and **Samir Khleif** (Augusta University, Augusta, USA) who have used a single dose of CPA of 50 mg before vaccination, which is similar to that used before T-cell adoptive transfer therapy.

The use of CPA in priming the immune response and suppressing the regulatory network was a major focus in the final discussion section of the meeting, with support from many speakers and participants that the data for enhancing immune responses were overwhelming, although the dose and schedules were highly variable and that there were significant problems in extrapolating from mice to humans, particularly with regard dose. It was concluded that there is a real need to directly compare pre-treatment with 300 mg iv with weekly or 1 week on and 1 week off metronomic therapy with CPA.

## Chemotherapy and myeloid-derived suppressor cells (MDSCs)

The importance of MDSCs in the development and maintenance of cancer growth and the need to inhibit them to switch off their marked immunosuppressive effects were mentioned as a major topic by a number of speakers, including **Michael Shurin** (University of Pittsburgh, Pittsburgh, USA), **Viktor Umansky** (Deutsches Krebsforschungszentrum, Heidelberg, Germany) and **Cornelis Melief** (Leiden University Medical Center, Leiden, Netherlands). **M Shurin** pointed out that MDSCs produced a range of oxidative molecules, which alters the TME. In particular, he pointed out that this degrades chemotherapies, such as doxorubicin, and that this effect needs to be protected to be effective as an anti-tumour agent. He presented a model using carbon nanotubes to encapsulate doxorubicin to aid delivery to the tumour in a B16 cell model. He proposed that encapsulated drugs, such as Taxol, could target MDSCs in particular. In previous meetings, **M Shurin** had emphasised the benefit of low-dose and ultralow-dose chemotherapies in different models in addition to the benefit of low-dose radiotherapy schedules. It is speculated that the observed benefit of chemotherapy may largely come from its effect on the TME and suppression of MDSCs, in particular.

**Rolf Kiessling** (Karolinska Institutet, Stockholm, Sweden) also focused on the importance of reducing MDSCs and showed that anti-cytotoxic T-lymphocyte-associated antigen 4 (anti-CTLA4) antibodies can reduce the numbers of MDSCs and T-regs, and especially the numbers of monocyte MDSCs in responding patients. This leads to an increase in CD8 effector memory cells in long-term survivors in patients with advanced melanoma. **Viktor Umansky** also focused on the importance of addressing MDSCs as a vehicle of immunosuppression, which is driven by chronic inflammation. He showed that there are two main types of MDSCs in humans, CD15+/CD14− polymorphonuclear neutrophils (PMN) MDSCs and CD15−/CD14+ monocytic MDSCs. They all express PD-L1 and their activation is influenced by the TME-inducing cytokines and exosomes derived from the tumour. Importantly, he showed that sildenafil inhibits cGMP activity, which normally induces NO, which effects NO reduction in mice. This translates to a survival benefit mediated by CD8 T cells, which recover ζ (zeta)-chain expression, IL-2 production and proliferation of T cells. In a human phase II study in pre-treated melanoma patients, tadalafil (a PDE-5 inhibitor) induced stable disease in 3/12 patients, who showed higher numbers of CD8 and TILs in their metastases and both CD4 and CD8 cells in their peripheral blood, increased CD3ζ-chain expression.

**Cornelis Melief** reported on the need to address the MDSCs in the blood and tumour to enhance vaccine effectiveness and that combining the HPV16-SLP vaccination protocol with chemotherapy (carboplatin and paclitaxel) gradually led to a reduction of MDSC and enhanced and sustained T-cell responses. He also mentioned that checkpoint inhibitors could further enhance these effects.

## Novel targets and strategies to reduce tumour immune suppression

**Jorg Wischhusen** (University of Würzburg, Würzburg, Germany) presented his work on growth differentiation factor (GDF)-15 (MIC-1) as a tumour immune-suppressive factor and that it could be an effective target for treatment. It is a divergent member of the TGFβ family, expressed at high levels in the placenta, prostate and then in stressed tissues. However, high levels are seen in melanoma patients, where high levels are an independent predictor of poor survival. GDF-15 reduces binding of ICAM to lymphocyte function-associated antigen-1 (LFA-1) thereby inhibiting adhesion to the endothelial cells. GDF-15 levels were also shown to predict poor response to anti-PD1 treatment. Data showing that anti-GDF-15 antibodies can enhance the effects of immunotherapy in mouse models support further development and translational studies in the clinic.

**Samir Khleif** reported on the ability of mutant genes, like KRAS, to influence the TME. Colorectal cancer patients with KRAS mutations have a greater number of T-regs. He presented data to show that FoxP3 expression correlated with mutant RAS and that if RAS is ‘knocked down’ there is a reduction in IL-10 and TGFβ. The practical application of this destruction is that ras signalling pathways can be targeted to enhance the immune response. He focused on the functional dichotomy in Class IA PI3K isoforms. The PI3Kδ (delta) was reported to be selective for T-reg function. A specific inhibitor of this pathway was shown to enhance the effectiveness of a tumour-specific vaccine by decreasing T-reg T cells and enhancing CD8 T cells. He then reviewed that CPA at low doses could also enhance this activity, which was the focus of his talk at the previous meeting.

**Thomas Sayers** (National Cancer Institute, Frederick, USA) reviewed his work on the molecular basis of apoptosis in tumours and the members of the TNF-related apoptosis-inducing ligand (TRAIL)-R family containing both pro- and anti-apoptotic receptors. Screening for agents that would sensitise tumour cells to apoptosis, he identified compounds from the withanolide family (derived from *Physalis peruviana*). Members of this family sensitise the tumour cells to TRAIL and DR4 and DR5 signalling. In addition, some cancer cells were also sensitised by withanolides to undergo apoptosis in response to the viral mimetic poly-(I:C). Mechanisms appear to depend on c-FLIP reduction by withanolides, which is a post-translational effect, there being no effect on gene expression. The relevant question to this meeting is ‘do these agents sensitise cells to become apoptotic when exposed to immunotherapy?’ In a murine B16 model he showed that the combination was superior to either agent alone but there is, as yet, no clinical translation to support this preliminary observation.

Other ways to enhance the response to immunotherapy with novel or repurposed drugs included the use of Zometa to enhance Vδ2^+^ γδ T-cell perforin-dependent cytotoxicity and how this relates to bone metastasis control. Details of this mechanism were presented by **Daniel Fowler** (St George’s University of London, UK) who specifically reviewed the effect on both M1 and M2 macrophages looking for a potential selective effect that could be exploited clinically.

**Barbara Seliger** (Martin-Luther-Universität, Halle-Wittenberg, Germany) extensively reviewed the reasons why MHC class I surface expression is reduced in tumours. This could be due to transcriptional, epigenetic and post-transcriptional regulation of MHC class I antigens or components of the antigen processing and presentation machinery. Furthermore, gemcitabine as well as IFN-γ have previously been reported to be able to reverse this impaired expression. She presented data that HDAC inhibitors can increase MHC class I expression in tumours of distinct origin. In addition, she showed the importance of not only the IFN-γ signalling pathway, in particular, the molecules JAK-1, JAK-2, STAT-1 for constitutive, but also IFN-γ-inducible MHC class I expression. Structural alterations of JAK-2 in melanoma cells leading to reduced MHC class I APM expression and resistance to IFN-γ treatment were detected and overexpression of JAK-2 into JAK-2-deficient cells restored IFN-γ sensitivity and enhanced constitutive MHC class I surface expression in these cells.

## Cancer vaccine updates

A major focus of this meeting was to review the latest in cancer vaccine development. **Lisa Butterfield** (University of Pittsburgh, Pittsburgh, USA) reviewed the enormous literature involving over 200 DC vaccine clinical trials in melanoma. Although the clinical responses are in the 5–10% range, there is no overall consistent effect and little correlation to vaccine immune responses. Essentially, we do not yet really understand the best method of maturation or loading, route of injection, best dose, etc. She briefly reviewed two previous DC trials, namely the MART-1 peptide pulsed on immature DCs and second, the DCs loaded with MART-1-expressing adenovirus. The main conclusion from these was that epitope spreading was associated with the best clinical responses, an effect which has also been reported with Dendreon’s Provenge studies. This suggests that targeting multiple antigens is better and IFNα may improve this effect. However, a multi-antigen-expressing adenovirus vector-transduced DC vaccine showed only 2 partial responses in 35 patients (neither of whom received IFNα). Microarrays of the DC vaccines, together with blood and tumour profiling studies, are being used to determine correlates of response to determine future combination therapies. There are currently five phase III and five phase II vaccines with checkpoint inhibitors (anti-PD-1) in progress.

This underscores a major ongoing trend, i.e. the use of PD-1 inhibitors in combination with other vaccines and immunotherapy agents to enhance clinical responsiveness. Whereas there is little to go on, in ways to optimise DC-based vaccines, **Brendon Coventry** (University of Adelaide, Adelaide, Australia) reported that vaccine responses are least likely to occur when the C-reactive protein **(**CRP) level is high and respond much better when it is low. He has previously reported that this level fluctuates cyclically even in individual patients. **Angus Dalgleish** (St George’s University of London, London, UK) has previously reported that the only correlate of clinical outcome observed in a previous DC-based vaccine for advanced melanoma patients was that responders were the only ones not to have elevated inflammatory markers. This has given rise to measuring these markers so that vaccines are given at the lowest levels (**B Coventry**) or to the realisation that perhaps anti-inflammatory agents may prime for vaccine responses, which has previously been shown in murine models.

**Victoria Brentville** (Scancell Ltd, Nottingham, UK) gave an update on the ImmunoBody® platform-based DNA vaccines which can induce high avidity T-cell responses to tumour-associated antigens (TAAs). Epitopes are engineered into the CDR3 region of immunoglobulin G (IgG). This is a whole antibody including the Fc region, which is absolutely necessary for function. The first clinical product is SCIBI, which has three epitopes from gp100 and one from TRP2 engineered into its CDR region. The vaccine alone has given a survival benefit in melanoma models (and patients), in combination with PD-1 antibodies, this effect is enhanced in murine models.

Their alternative approach depends on identifying suitable siPTMs and boosting them through a platform called moditope. This work has focused on the autophagy pathway for long-lived proteins which cause citrullination of arginine by Ca^2+^-dependent peptidylarginine deiminase (PAD) enzymes. This has led to the development of vimentin (VIM)-based citrullinated peptide-based vaccines that have been shown to be very effective in mouse models. A lead clinical target containing multiple such peptides is being developed.

**Bernard Fox** (Earle A Chiles Research Institute, Portland, USA) presented his data using a similar but different approach to creating a novel multivalent vaccine by disrupting the degradation of intracellular proteins by the ubiquitin proteasome system. This also involves autophagosome products and is branded as the DRibbles vaccine, containing DRiPs (defective ribosomal products) and SLiPs (short-lived proteins), including TAAs. This involves blocking and stabilising these agents and harvesting autophagosomes by membrane disruption and fractionalisation to create an ‘autologous vaccine’. Pre-clinical work looked at the effect of these vaccines on the expression of γIFN in the draining lymph nodes, showing that the DRibbles approach was much better than a whole cell vaccine in enhancing γIFN expression.

Combining this approach with anti-OX40 antibodies enhanced protection in mice, which led to looking at an allogenic preparation of DRibbles for large-scale studies (DPV-001). A randomised study for stage III/IV NSCLC patients is underway with CPA being used as an immune modulator. Combination with other checkpoint inhibitors (e.g. PD-1) is another obvious possibility.

It is also of relevance here to highlight the content of the winning poster presentation by **Derin B Keskin** (Harvard Medical School, Boston, USA). Previous methodologies for the prediction of peptide presentation have had limitations and were subject to certain biases. Derin B Keskin presented methodology based on high-resolution liquid chromatography–mass spectrometry (LC–MS/MS) which was used to identify HLA-allele-specific peptides. These peptides were used to train new algorithms which can be used to predict HLA-binding epitopes with more accuracy. This poster also detailed use of a relatively new technology (non-scale MS) which is being developed for the identification of neo-epitopes in tumour biopsy material.

**Alan Melcher** (Institute for Cancer Research, London, UK) reviewed the potential of oncolytic viruses to enhance the immune response. T-VEC, an HSV-1-modified agent expressing GM-CSF gene, has been licensed for intratumoural administration in melanoma and has been shown to induce regression of both injected and non-injected lesions. Once again, this effect has been enhanced by combining it with checkpoint inhibitors. He also reviewed the potential for replication competent reovirus to enhance specific anti-tumour responses by making ‘cold’ tumours (low CD8+ TILs/PD-L1 expression) ‘hot’, leading to tumour regression when combined with PD-1 blockade.

This was a consistent theme of the meeting in that PD-1 antibodies only work in less than 50% of the patients but they may be much more effective if tumours are primed with vaccines, etc. to make them ‘hot’.

Another theme of the meeting was the benefit of drugs that can immunomodulate the immune response and several, such as CPA, gemcitabine and anti-inflammatories, have already been mentioned. **Rajesh Chopra** (Institute for Cancer Research, London, UK) reporting on the most effective immune modulators described the immunomodulatory drugs (IMiDs), which include Revlimid (lenalidomide) and pomalidomide. He reviewed the insight into the mechanisms of action following the discovery of the interaction with cereblon (CRBN) as a target for thalidomide. There are several areas for new agents from this work, such as targeting the unfolded protein response, such as the IRE–XBP-1 pathway. He also reviewed the importance of targeting the endoplasmic reticulum aminopeptidase-1 (ERAP1) and -2 pathways, which can lead to more immunogenic and larger peptides. Knockout ERAP1 mice gave a survival benefit over controls. This work is complimentary to that of **C Melief**, who has demonstrated the benefit of long HPV peptide-based vaccines clinically. There is an obvious potential for combination of these approaches in the future.

## Neuroblastoma antibody therapy

**Paul Sondel** (University of Wisconsin, Madison, USA) has helped pioneer the use of antibodies to ganglioside molecule-2 (GD2) in patients with neuroblastoma, which has taken 20 years to develop phase III trials, leading to approval by the FDA and EMA. A component of this regimen is the combination with IL-2, which activates NK cells and GM-CSF, which activates neutrophils and monocyte/macrophages, all of which may be helpful via antibody-dependent cell-mediated cytotoxicity (ADCC). Unfortunately, the treatment still does not work in some patients and attempts to develop a good biomarker, via killer cell receptors (KIR)/KIR–ligand genotyping, were described. A humanised anti-GD2 with IL-2 engineered at the Fc terminal has now been developed. In murine models, this can be enhanced by intratumoural delivery and anti-checkpoint inhibitors (anti-CTLA-4). Radiotherapy (RT) could enhance this effect when given prior to treatment.

**Emily Webb** is also investigating this approach for neuroblastoma and addressing the fact that relapse is common. Using animal models (NXS2) enhancement was shown with CPA, which depleted T-regs, as well as combining with anti-PD-L1 treatments. The outcome of these presentations strongly suggests an optimisation of anti-CD-2 antibody with CPA, IL-2 and anti-PD-L1 antibodies.

## Combination antibodies

Combining antibodies was the focus of Holbrook Kohrt, who presented this approach at our previous meeting. Sadly, he died from the effects of haemophilia.

**Ignacio Melero** reviewed the potential of this approach using a car analogy: the need to take the foot off the break, step on the gas and pave the road. Interest is growing in alternative checkpoint blockade targets. He focused on anti-CD137 that Holbrook had identified, and which is already known to enhance rituximab in multiple myeloma. In mice, he showed how a combination of anti-41BB (the murine homologue of CD137) with anti-PD-1 had anti-tumour effects. These effects were not observed in the Batf3-deficient mouse suggesting an importance of CD103+ dendritic cells. **I Melero** further demonstrated an abscopal effect in mouse where treatment with radiotherapy in combination with anti-PD-1 led to response of distant lesions as well as locally treated lesions.

## Practical aspects and future studies

It is now so clear that combination of these approaches should be more effective that singular approaches. Unfortunately, this introduces major issues with regard to increased toxicity, increased clinical development times and, most importantly, increased costs, which may make such logical approaches prohibitively expensive. **Sandra Tuyaerts** described plans for a revolutionary new study which aims to treat cervical, endometrial and uterine sarcoma with a combination based on many of the mechanisms already reviewed here. It aims to combine PD-1 blockade, radiation and several agents known to have favourable immune modulatory properties, such as CPA, lansoprazole, aspirin and vitamin D3. Assessment of the study primary endpoint will be performed using RECIST and immune-related response criteria at 26 weeks.

**Thomas Nesselhut** has long since expressed his views that carefully controlled observations can guide optimal immunotherapy development. (It is worth remembering that multiple chemotherapy for testes and lymphoma treatment evolved without randomised studies!) He presented a number of cases, emphasising the benefit of enhancing the efficacy of his autologous DC-based vaccines using CPA (which agreed with many of the other presenters) the use of Newcastle disease virus (NDV) to prime DC vaccines, along with hyperthermia. (NDV is an oncolytic virus and hence this fits with data presented by **A Melcher**.) More recently, he has also been using NDV with Zometa plus gamma–delta T-cell treatment, which is entirely consistent with the data presented by **Dan Fowler**. He has also used low-dose naltrexone (LDN) as an immune stimulator, as has Julian Kenyon, who commented on its benefit in hundreds of patients he has treated anecdotally. This was of great interest as **Rachel Cant** (St George’s University of London, UK) reported on antagonism of TLRs 7, 8, and 9 by naltrexone, which could help explain the anti-inflammatory and immune stimulatory effects of LDN that are hard to explain based on its opiate receptor interaction above.

## Concluding remarks

Many of these recurrent themes were discussed in detail in the final session, with a clear consensus on the need to optimise immunotherapy combinations and immune modulatory effects of chemotherapy, especially with regard to the use of CPA, which was mentioned by several speakers. The importance of biomarkers to help decide treatment options was agreed as important by many.

**Bernard Fox** raised the important potential of the microbiome and the effects of chemotherapy on gut inflammation and microbiome, as this is the focus of intense research by several established investigators.

Overall, the discussion paid homage to the memory of Holbrook Kohrt and his dramatic contribution to the field and pioneering contributions to combination immunotherapy. In memory of this it was proposed that his legacy be honoured with a named lecture. Many people were asked to nominate the first Holbrook Kohrt lecture and the fact that both Bernard Fox and Paul Sondel came out on tie led to the decision to invite both of them to give named lectures in his honour.

## Abbreviations

ADCC: Antibody-directed cell-mediated cytotoxicity
Batf3: Also known as Jun dimerization protein p21SNFT
CVI: Cancer Vaccine Institute
c-FLIP: Cellular FLICE-like inhibitory protein
CDR-3: Complement-determining region no. 3
CPA: Cyclophosphamide
CRBN: Cereblon
CRP: C-reactive protein
DRiPs: Defective ribosomal products
EMA: Euopean Medical Agency
ERAP: Endoplasmic reticulum aminopeptidase
FDA: Food and Drug Administration
GD2: Ganglioside molecule
GDF15: Growth/differentiation factor 15
HDAC: Histone deacetylases
ICVI: Institute for Cancer Vaccines and Immunotherapy
IMiDs: Immunomodulatory drugs
Ire–xbp: Iron regulatory protein–x box protein
KIR: Killer cell receptors
LC–MS: Liquid chromatography–mass spectrometry
LDN: Low-dose naltrexone
LFA-1: Lymphocyte function-associated antigen-1
MART: Melanoma-associated antigen recognised by T cells
MHC: Major histocompatibility complex
MIC-1: Macrophage inhibitory cytokine 1
NDV: Newcastle disease virus
PAD: Peptidylarginine deiminase
PDE: Phosphodiesterase
PMN: Polymorphonuclear neutrophils
RFA: Radiofrequency ablation
RT: Radiotherapy
SiPTM: Stress-induced post-translational modifying
SLiPs: Short-lived proteins
SLP: Synthetic long peptide
TRP2: Tyrosinase-related protein 2
T-VEC: Talimogene laherparepvec
VIM: Vimentin

